# The usage of biological DMARDs and clinical remission of rheumatoid arthritis in China: a real-world large scale study

**DOI:** 10.1007/s10067-016-3424-5

**Published:** 2016-10-05

**Authors:** Yuan An, Tian Liu, Dongyi He, Lijun Wu, Juan Li, Yi Liu, Liqi Bi, Bin Zhou, Changsong Lin, Lan He, Xiangyuan Liu, Xiaofeng Li, Niansheng Yang, Zhuoli Zhang, Hui Song, Wei Wei, Jing Liu, Yu Bi, Zhanguo Li

**Affiliations:** 1 Department of Rheumatology and Immunology, Peking University People’s Hospital, 11 South Xi Zhi Men Street, Xicheng District, Beijing, 100044 People’s Republic of China; 2 Department of Rheumatology, Shanghai Guanghua Hospital, Shanghai, China; 3 Department of Rheumatology, People’s Hospital of Xinjiang Uygur Autonomous Region, Xingjiang, China; 4 Department of Rheumatology, Nanfang Hospital of Southern Medical University, Guangzhou, China; 5 Department of Rheumatology and Immunology, West China Hospital, West China School of Medicine, Sichuan University, Chengdu, China; 6 Department of Rheumatology, China-Japan Union Hospital of Jilin University, Changchun, China; 7 Department of Rheumatology, Sichuan Provincial People’s Hospital, Sichuan, China; 8 Department of Rheumatology, The First Affiliated Hospital of Guangzhou Traditional Chinese Medicine University, Guangzhou, China; 9 Department of Rheumatology, The First Affiliated Hospital of Xi’An Jiaotong University, Xi’an, China; 10 Department of Rheumatology, Peking University Third Hospital, Beijing, China; 11 Department of Rheumatology, The Second Hospital of Shanxi Medical University, Taiyuan, China; 12 Department of Rheumatology, The First Affiliated Hospital of Sun Yat-sen University, Guangzhou, China; 13 Department of Rheumatology, Peking University First Hospital, Beijing, China; 14 Department of Rheumatology, Beijing Jishuitan Hospital, Beijing, China; 15 Department of Rheumatology, Tianjin Medical University General Hospital, Tianjin, China; 16 Shanghai Roche Pharmaceuticals Ltd, Shanghai, China

**Keywords:** Biological, China, DMARD, Duration, Rheumatoid arthritis, Therapy

## Abstract

The aims of this study are to characterize the biological disease-modifying antirheumatic drug (bDMARD) usage patterns in real-life and examine the remission rate of rheumatoid arthritis (RA) patients receiving bDMARDs in routine clinical practice in China. Consenting RA patients (≥18 years) from 15 teaching hospitals and receiving marketed bDMARDs were included. In total, 802 patients (81.3 % women, 49.0 ± 13.9 years) were included; 89.5 % were receiving a combination of bDMARDs and conventional synthetic DMARDs (csDMARDS), whereas 10.5 % were receiving bDMARD monotherapy. Etanercept (including Enbrel® and local brand Yi Sai Pu® and Qiangke®), tocilizumab, adalimumab, and infliximab were used by 66.6 %, 17.0 %, 7.5 %, and 6.6 % patients, respectively. Etanercept was used at a mean weekly dose of 38.2 ± 15.6 mg for 25.5 ± 47.0 weeks and tocilizumab at 94.5 ± 21.9 mg for 4.7 ± 7.5 weeks. Overall rate of remission was 12.6 %, 5.4 % , and 3.5 % based on DAS28, CDAI, and SDAI scores, respectively. Compared with patients receiving bDMARDs for <3 months, those receiving bDMARDs for ≥3 months exhibited significantly lower DAS28 scores (*p* < 0.0001), and a significantly higher proportion of patients who received bDMARDs for ≥12 months achieved the treatment goal (remission or low disease activity, 62.5 % vs. 18.3 %, *p* < 0.0001). Patients receiving combination therapy with csDMARDs exhibited lower DAS28 scores than patients receiving bDMARD monotherapy (4.3 vs. 4.8, *p* = 0.011). This large-scale real-world study showed that bDMARD usage patterns in routine clinical practice in China were in accordance with international guidelines for RA management despite the short treatment duration. Longer duration of bDMARD usage and combination therapy showed a favored outcome of RA.

## Introduction

Rheumatoid arthritis (RA) is a chronic systemic autoimmune disease associated with progressive joint damage and disability. The prevalence of RA is 0.24 % globally and between 0.3 and 1 % in developed countries [1, 2]. In Mainland China, the prevalence of RA has been reported to be 0.28 % [3, 4]. As shown by recent studies, RA is becoming one of the most common disabling and costly disease in China [5–7]. Chou et al. reported a significantly higher RA prevalence in urban areas than in rural areas of Taiwan [8]. Socioeconomic and genetic factors might contribute to the lower prevalence in China compared with Western countries [9].

Disease-modifying antirheumatic drugs (DMARDs) are the standard treatment for patients with RA [10]. In addition to conventional synthetic DMARDs (csDMARDs), such as methotrexate, biological DMARDs (bDMARDs), including inhibitors targeting tumor necrosis factor (TNF)-α, T cells, B cells, and interleukin-6, have been increasingly used in recent years [10, 11]. According to the 2013 European League Against Rheumatism (EULAR) and the 2012 American College of Rheumatology (ACR) recommendations, csDMARDs such as methotrexate should be chosen as the first-line treatment [10, 11]. However, the recommendations suggest bDMARDs for patients who fail to achieve low disease activity (LDA) or remission after receiving csDMARDs [10, 11]. Although a combination therapy of csDMARDs and bDMARDs is recommended and commonly practiced, real-life registry data show that approximately 30 % of patients receive bDMARDs as monotherapy in Western countries [12]. In a double-blind randomized trial, Dougados et al. found that the efficacy of tocilizumab monotherapy was comparable to a combined therapy of tocilizumab plus methotrexate in patients who had insufficient responses to methotrexate [13]. Emery et al. systematically reviewed trials evaluating bDMARD monotherapy and found that only tocilizumab monotherapy appeared to show efficacy equivalent to combination therapy with methotrexate [12]. In a recent large observational study, Gabay et al. found that patients receiving tocilizumab with or without concomitant csDMARD showed comparable clinical response [14].

Until the end of patient enrollment of this study, a total of six bDMARDs have been approved for use in RA in China, including the interleukin-6 receptor inhibitor, tocilizumab, and five TNF inhibitors (infliximab, adalimumab, and etanercept, including Enbrel® and local brand Yi Sai Pu® and Qiangke®). In recent years, bDMARDs have been increasingly prescribed in routine clinical practice in China. However, the “real-world” data on bDMARD usage patterns in China might be different from those in developed countries owing to socioeconomic factors. The objective of this cross-sectional study was to characterize the usage patterns of bDMARDs in Chinese patients with RA and examine the association between disease activity and clinical remission, and the duration and pattern of bDMARD therapy, with the goal to optimize the management of RA in China.

## Materials and methods

### Study design

This multicenter observational cross-sectional study was conducted in 15 hospitals across different regions of China. The study was approved by the Institutional Review Board of Peking University and independent ethics committees responsible for each investigating site. The study was conducted in compliance with the Declaration of Helsinki and in accordance with Good Clinical Practice and relevant ethical guidelines. Written informed consent was obtained from each participant.

### Participants

Patients aged ≥18 years with confirmed RA according to the ACR 1987 criteria [15] and receiving bDMARDs at the time of interview were enrolled in the study. Patients were excluded if their physicians believed that the patients were not appropriate to participate in the study. Non-consenting patients were excluded.

### Data collection

Questionnaires were administered through face-to-face interviews in each center by trained participating investigators with eligible patients. Data on age, sex, RA duration, total joint count, swollen joint count, and visual analogue scale (VAS) were collected on enrollment visit. The levels of C-reactive protein, hemoglobin, erythrocyte sedimentation rate, rheumatoid factor, and anti-cyclic citrullinated peptides measured within 2 weeks of the enrollment visit were obtained from medical records. Disease activity was evaluated according to the Disease Activity Score based on 28-joint count (DAS28) (remission: DAS28 < 2.6; low disease activity: 2.6 ≤ DAS28 < 3.2; moderate disease activity: 3.2 ≤ DAS28 < 5.1; high disease activity: DAS28 ≥ 5.1 [16, 17]), the Simplified Disease Activity Index (SDAI) (remission: SDAI ≤3.3; low disease activity: 3.3 < SDAI ≤11; moderate disease activity: 11 < SDAI ≤26; high disease activity: SADI >26), and the Clinical Disease Activity Index (CDAI) (remission: CDAI ≤2.8; low disease activity: 2.8 < CDAI ≤10; moderate disease activity: 10 < CDAI ≤22; high disease activity: CDAI >22).). Quality of life of patients was assessed using health assessment questionnaire disability index (HAQ-DI) on enrollment visit. Visual analog scales on enrollment visit (from 0 to 10 cm) were used to assess fatigue and pain levels, with increasing values representing more severe fatigue and pain.

Data on usage patterns of bDMARDs, including information on monotherapy or combination therapy, the drug(s) used, frequency, treatment duration, and dosage of bDMARDs, were collected. The reasons for discontinuation or switching of bDMARDs and information about previous bDMARDs, including drug name and treatment duration, were recorded. All the AE information of using the common biologic agents was recorded and followed until return to stabilized.

### Sample size estimation

The formula to calculate sample size in a prevalence survey was used [18, 19]. Based on European and US registry data, approximately one third (33 %) of RA patients receive bDMARDs as monotherapy. Thus, with the assumption that the proportion of patients in China receiving bDMARD monotherapy is also 33 % and a confidence level of 95 % with maximum relative error of 10 %, the required sample size was estimated to be 800.

### Statistical analysis

The statistical analysis was performed using the statistical software SAS 9.2 (Cary, North Carolina, USA). Continuous data are presented as mean and standard deviation (SD), median, and range. Two-group comparisons were performed using Student *t* test (*p* values were two-sided with a significance level of 0.05). Multiple-group comparisons were performed by analysis of variance with Bonferroni correction. Laboratory tests performed in different hospitals were standardized by statistical adjustment [20]. Categorical data, tabulated as frequencies and percentages, were analyzed using chi-square test. Missing values were excluded from the analyses.

## Results

### Patient characteristics and bDMARD usage patterns

A total of 808 patients were enrolled between December 2013 and August 2014. Six patients were excluded for violating the inclusion criteria (five patients were diagnosed with ankylosing spondylitis and one patient was <18 years old). Available data from 802 patients were analyzed. As shown in Table 1, patients (mean age of 49.0 ± 13.9 years) had a mean disease course of 3.2 ± 5.8 years. Abnormal C-reactive protein and erythrocyte sedimentation rate levels were exhibited by 60.8 % and 70.1 % of patients, respectively. The majority of the patients were positive for rheumatoid factor (77.6 %) or anti-cyclic citrullinated peptides (83.2 %). Disease activity in the patients varied widely, as reflected by a broad range of DAS28, CDAI, and SDAI scores. The patients reported good quality of life and medium levels of fatigue and pain.

**Table 1:** Patient characteristics (*N* = 802)

	Patients, *n* (%)	Mean (SD)	Median (range)
Age (year)	800	49.0 (13.9)	50.3 (18.2–84.2)
Gender, *n* (%)			
Men	150 (18.7)		
Women	652 (81.3)		
Body weight (kg)	799	58.9 (10.6)	58.0 (30.0–104.0)
RA status			
Disease course^a^ (years)	357	3.2 (5.8)	0.6 (0.0–40.3)
0.0–0.5, *n* (%)	176 (49.3)		
0.5–10, *n* (%)	139 (38.9)		
More than 10, *n* (%)	42 (11.8)		
CRP (mg/mL)	344	27.7 (33.9)	12.7 (0.1–210.0)
Abnormal^b^, *n* (%)	209 (60.8)		
ESR (mm/hour)	394	42.4 (31.1)	35.5 (1.0–140.0)
Abnormal^b^, *n* (%)	276 (70.1)		
Hemoglobin (g/L)	411	117.1 (18.8)	118.3 (63.7–165.2)
Anemia^c^, *n* (%)	147 (35.8)		
RF, *n* (positive %)	184 (77.6)		
ACCP, *n* (positive %)	144 (83.2)		
DAS28	412	4.4 (1.5)	4.4 (0.5–7.7)
CDAI	801	20.2 (15.3)	16.0 (0.0–76.0)
SDAI	343	27.2 (18.1)	23.9 (0.13–83.5)
TJC	802	6.7 (7.2)	4.0 (0.0–28.0)
SJC	802	4.8 (6.1)	2.0 (0.0–28.0)
Quality of life assessment			
HAQ-DI	801	11.9 (13.4)	7.0 (0.0–60.0)
VAS-fatigue	801	3.7 (2.5)	3.5 (0.0–10.0)
VAS-pain	801	4.2 (2.4)	4 (0.0–10.0)

*SD* standard deviation, *RA* Rheumatoid arthritis, *CRP* C-reactive protein *ESR* Erythrocyte sedimentation rate, *RF* Rheumatoid factor, *ACCP* Anti-cyclic citrullinated peptide, *DAS28* Disease activity score based on 28-joint count, *CDAI* Clinical disease activity index, *SDAI* Simplified disease activity index, *TJC* Tender joint count, *SJC* Swollen joint count, *HAQ-DI* Health assessment questionnaire disability index, *VAS* Visual analog scale

^a^Disease course was calculated according to the following equation: (the date when a patient signed the informed consent − the data when RA was diagnosed + 1) / 365.25

^b^The definitions of abnormal CRP and ESR follow participating hospitals’ standardized criteria

^c^Anemia was defined as hemoglobin <120 g/L for men and hemoglobin <110 g/L for women

In the current study, etanercept was used by 66.6 % (including Yi Sai Pu® 58.1 %, Enbrel® 6.1 %, and Qiangke® 2.4 %) patients. Tocilizumab, adalimumab, and infliximab were used by 17.0 %, 7.5 %, and 6.6 % of patients, respectively. The mean weekly doses and durations of bDMARDs are shown in Table 2. Only 10.5 % of patients were receiving bDMARD monotherapy, amongst who, 75.0 %, 10.7 %, 9.5 %, and 4.8 % were using etanercept, infliximab, tocilizumab, and adalimumab, respectively. The remaining patients (89.5 %) were receiving combination therapy of csDMARDs and bDMARDs. Most patients received one (49.3 %) or two (41.2 %) csDMARDs, whereas only 9.5 % were on three csDMARDs. Among the patients on combination therapy, the proportion of patients using etanercept, infliximab, tocilizumab, or adalimumab was 65.7 %, 8.4 %, 18.1 %, and 7.8 %, respectively; these proportions were similar to those of patients on bDMARD monotherapy. The dose and duration of bDMARDs in combination therapy were comparable to those in bDMARD monotherapy (Table 3). The most commonly administered concomitant csDMARD was methotrexate, used by 65.9 % of patients at a mean weekly dose of 9.8 ± 2.8 mg for 63.4 ± 120.8 weeks. Furthermore, 41.8 % and 41.5 % of patients were using concomitant hydroxychloroquine (2382.1 ± 674.1 mg/week) and leflunomide (107.8 ± 36.4 mg/week), respectively. In addition to csDMARDs, other types of drugs, including nonsteroidal anti-inflammatory drugs (NSAIDs), glucocorticoids, and topical drugs, were concomitantly used by 56.1 %, 29.7 %, and 19.1 % of patients, respectively (Table 3).

**Table 2:** Dose and treatment duration of bDMARD therapy

bDMARD	Patients, *n* (%)	Weekly dose (mg)		Treatment duration (weeks)	
		Mean (SD)	Median (range)	Mean (SD)	Median (range)
Etanercept	534 (66.6)	38.2 (15.6)	50 (2.1–100.0)	25.5 (47.0)	6.0 (0.1–350.0)
Tocilizumab	136 (17.0)	94.5 (21.9)	100 (30.0–160.0)	4.7 (7.5)	1.0 (0.1–34.0)
Adalimumab	60 (7.5)	20.1 (6.4)	20.0 (5.0–40.0)	12.7 (19.4)	4.0 (0.1–104.0)
Infliximab	53 (6.6)	33.1 (23.6)	25.0 (3.8–100.0)	34.5 (39.1)	24.0 (0.1–186.0)

**Table 3:** bDMARD monotherapy and combination therapy (*N* = 802)

Therapy type	Patients *n* (%)	No. of patients with available therapy duration	Duration (week)	
			Mean (SD)	Median (range)
bDMARD monotherapy^b^	84 (10.5)^a^	84	27.4 (54.6)	1.5 (0.1–240.0)
Etanercept	63 (75.0)	63	29.0 (58.2)	1.0 (0.1–240.0)
Infliximab	9 (10.7)	9	42.0 (60.8)	24.0 (0.1–186.0)
Tocilizumab	8 (9.5)	8	8.0 (9.8)	4.1 (0.1–28)
Adalimumab	4 (4.8)	4	7.4 (13.7)	0.8 (0.1–28.0)
bDMARD + csDMARD combination therapy^c^	718 (89.5)^a^	718	21.1 (39.7)	4.4 (0.1–350.0)
Etanercept	472 (65.7)	472	25.0 (45.4)	6.0 (0.1–350.0)
Infliximab	60 (8.4)	60	33.3 (35.3)	23.0 (0.1–165.0)
Tocilizumab	130 (18.1)	130	4.5 (7.3)	1.0 (0.1–34.0)
Adalimumab	56 (7.8)	56	13.1 (19.8)	4.0 (0.1–104.0)
Number of concomitant csDMARDs	718 (100)			
One kind of csDMARDs	354 (49.3)		NA	NA
Two kinds of csDMARDs	296 (41.2)		NA	NA
Three kinds of csDMARDs	68 (9.5)		NA	NA
Type of concomitant csDMARDs^d^	718 (100)			
Methotrexate	473 (65.9)	468	63.4 (120.8)	16.0 (0.1–999)
Hydroxychloroquine	300 (41.8)	298	37.3 (58.5)	10.0 (0.1–364.0)
Leflunomide	299 (41.6)	296	59.1 (82.6)	28.0 (0.1–520.0)
Sulfasalazine	46 (6.4)	45	61.3 (148.6)	12.0 (0.1–750.0)
Others	34 (4.7)	32	NA	NA
Other concomitant drugs	802 (100)		NA	NA
Nonsteroidal anti-inflammatory drugs	450 (56.1)		NA	NA
Glucocorticoids^e^	238 (29.7)		NA	NA
Topical drugs	153 (19.1)		NA	NA

^a^Percentage was calculated using the total number of patients (*N* = 802) as the denominator

^b^bDMARD monotherapy represents bDMARD alone without combination with csDMARDs; the percentage of each bDMARD was calculated using the total number of patients on monotherapy (*n* = 84) as the denominator

^c^bDMARD + csDMARD combination therapy represents combination of bDMARDs and csDMARDs; the percentage of each bDMARD was calculated using the total number of patients on combination therapy (*n* = 718) as the denominator

^d^The percentage of each type of csDMARD was calculated using the total number of patients on bDMARD + csDMARD combination therapy (*n* = 718) as the denominator

^e^Among the 238 patients on concomitant glucocorticoids, 73.1 and 23.1 % patients were receiving oral prednisone and methylprednisolone at a mean (SD) weekly dose (mg) of 57.8 (31.9) and 59.7 (151.6), respectively

The three top reasons for discontinuing bDMARDs (*n* = 58) were clinical improvement (31.0 %), financial burden (24.1 %), and AEs (13.8 %). Among patients switching to different bDMARDs (*n* = 93), the main reasons for switching were unsatisfactory efficacy of the previous bDMARD (58.1 %), AEs (14.0 %), improvement of disease condition (10.8 %), and financial burden (10.8 %). Among the 802 patients, only 5 (0.6 %) reported at least one AE after initiation of this study, including one case of mild pruritus and another case of rash, which were suspected to be associated with etanercept. No bDMARDs-related serious AE was reported. Further analyses of disease activity revealed that the overall rate of remission was 12.6 %, 5.4 %, and 3.5 % based on DAS28, CDAI, and SDAI scores, respectively.

### Short duration of bDMARD therapy was associated with poor management of RA

**Fig. 1:**
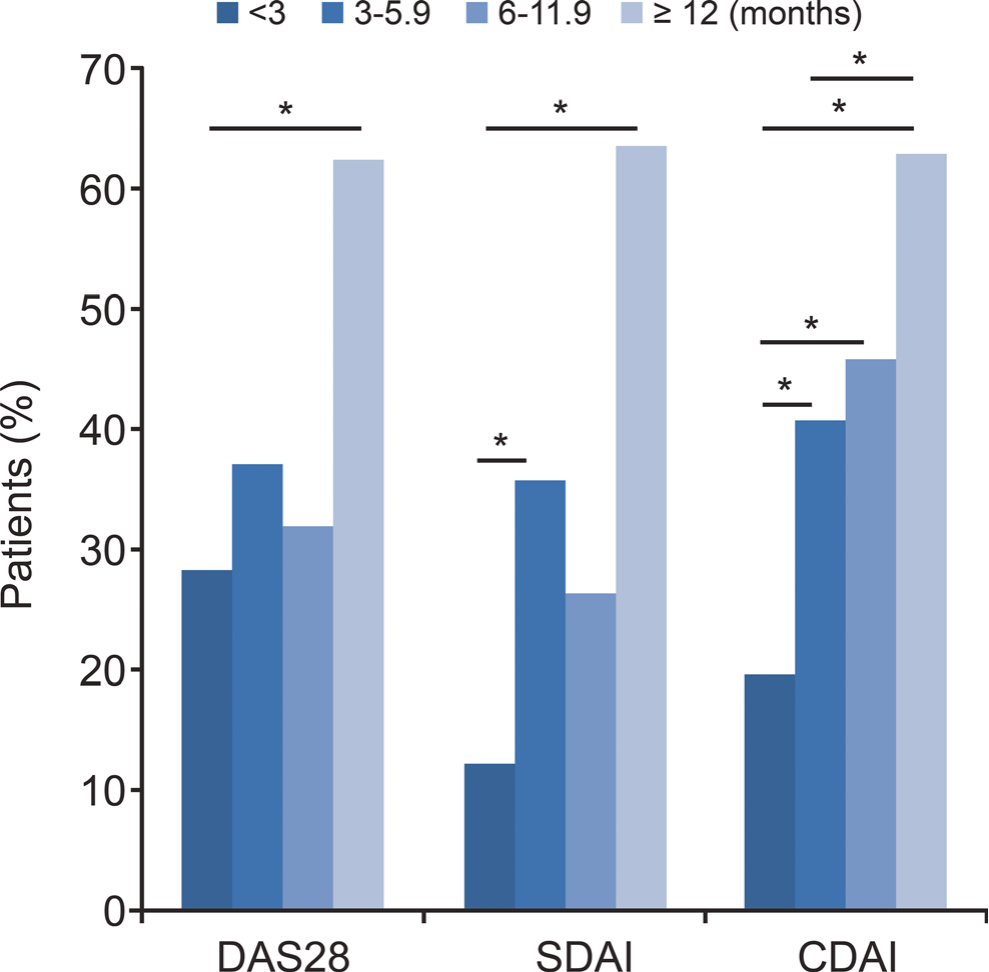
The proportion of patients achieving treatment goal with various durations of bDMARDs. Comparison was performed by multiple comparisons with Bonferroni adjustments (the adjusted *α* = 0.05/6 = 0.0083; *p < 0.0083)

The duration of bDMARD therapy in the patients varied from 0.1 to 350.0 weeks (Table 2). Patients receiving bDMARDs for 3.0–5.9 months or for >12 months had significantly lower DAS28 scores than those receiving bDMARDs <3 months (*p* = 0.0002 and *p* < 0.0001, respectively, Table 4). A significantly larger proportion of patients with ≥12 months bDMARD therapy (DAS28 62.5 %, SDAI 63.6 %, CDAI 62.9 %) achieved treatment target (LDA or remission) compared with patients with <12 months (DAS28 21.0 %, SDAI 15.2 %, CDAI 25.8 %, all *p* < 0.05). In contrast, the proportion of patients achieving treatment target was significantly lower in patients with <3 months bDMARDs (SDAI 12.2 %, CDAI 19.5 %) than in those with 3.0–5.9 months bDMARDs (SDAI 35.7 %, CDAI 40.7 %, all *p* < 0.0083, Fig. 1). The proportion of patients achieving treatment target was not significantly different between patients with 3–5.9 months bDMARDs and patients with 6.0–11.9 months (all *p* > 0.0083, Fig. 1).

**Table 4:** Association of bDMARD therapy duration and DAS28 scores

Treatment duration (months)	Patients^a^ (*n* = 412)	Mean score (SD)	*p* value
0.0–2.9	312	4.6 (1.5)	
3.0–5.9	35	3.5 (1.4)	0.0002*
6.0–11.9	25	3.9 (1.4)	0.0945*
>12	40	3.2 (1.4)	<0.0001*

*Compared with 0.0–2.9-month group, with analysis of variance followed by Bonferroni correction. The adjusted *α* = 0.05/6 = 0.0083; *p < 0.0083

^a^Number of patients with available score data

### The effects of treatment regimen on disease activity

Patients receiving combination therapy of bDMARDs and csDMARDs showed significantly lower mean DAS28 scores than patients on bDMARD monotherapy (4.3 vs. 4.8, *p* = 0.0108, Table 5). However, the percentage of patients achieving treatment target was not significantly different between bDMARD monotherapy and combination therapy (21.4 % vs. 25.6 %, *p* = 0.507, Table 5). Further examination of patients with bDMARD monotherapy revealed that DAS28 score was significantly lower in patients receiving tocilizumab than in patients receiving TNF inhibitors (3.6 ± 2.1 vs. 5.0 ± 1.7, *p* = 0.0479, Table 5). Consistently, significantly higher proportion of patients with tocilizumab monotherapy reached treatment target compared with patients receiving TNF inhibitor type of bDMARDs (57.1 % vs. 16.3 %, *p* = 0.0317, Table 5). Notably, the DAS28 score in patients receiving TNF inhibitors as monotherapy was significantly higher than that in patients receiving combination therapy of TNF inhibitors and csDMARDs (5.0 ± 1.7 vs. 4.3 ± 1.4, *p* = 0.0012, Table 5), suggesting that TNF inhibitor monotherapy may not be as effective as combination therapy of TNF inhibitors plus csDMARDs. In contrast, DAS28 score was not significantly different in patients receiving tocilizumab monotherapy and patients receiving combination therapy of tocilizumab and csDMARDs (3.6 ± 2.1 vs. 4.3 ± 1.6, *p* = 0.2672, Table 5).

**Table 5:** The effects of treatment type on DAS28 score and treatment target (low disease activity and remission)

	Patients^a^	Mean DAS28 score (SD)	*p* value	Remission + LDA rate (%)	*p* value
bDMARD therapy	412 (total)				
bDMARD monotherapy	56	4.8 (1.8)	0.0108	21.4	0.507
bDMARD combination therapy	356	4.3 (1.5)		25.6	
bDMARD monotherapy	56 (total)				
TNF inhibitors^b^	49	5.0 (1.7)	0.0479	16.3	0.0317
Tocilizumab	7	3.6 (2.1)		57.1	
TNF inhibitors^b^	327				
Monotherapy	49	5.0 (1.7)	0.0012		
Combination therapy	278	4.3 (1.4)			
Tocilizumab	85				
Monotherapy	7	3.6 (2.1)	0.2672		
Combination therapy	78	4.3 (1.6)			

^a^Number of patients with available score data

^b^TNF inhibitors include infliximab, adalimumab, and etanercept

## Discussion

This is the first large-scale multicenter study to characterize real-life bDMARD usage patterns and disease activity in routine practice in Chinese patients with RA receiving bDMARDs. The results show that combination therapy of bDMARDs and csDMARDs was substantially more common than bDMARD monotherapy in real-world clinical practice in China. Methotrexate was the predominant csDMARD administered to Chinese patients. The usage pattern of bDMARDs in China is in accordance with the guidelines for management of RA, in which methotrexate is recommended as the first-line agent and bDMARDs are used as secondary agents, mostly in combination with csDMARDs [10].

This study found that the proportion of patients receiving bDMARD monotherapy in China was only 10.5 %, considerably lower than that in other countries. Yazici et al. investigated the RA patient cohort in the Thomson Healthcare MarketScan Research databases and found that 30 % of adults with RA received bDMARD monotherapy in the USA [19]. Gabay et al. examined the data from the Swiss Clinical Quality Management in Rheumatic Diseases registry for RA patients and found that bDMARD monotherapy was prescribed for up to 39 % of treatment courses [21]. Kaufmann et al. conducted a retrospective study of 254 German patients and found that between 18 % and 41 % of patients treated with bDMARDs received the agent as monotherapy [22].

Overall, disease control in our patient cohort was not optimal despite the use of bDMARDs. The majority (75 %) of patients were classified as having moderate- or high-disease activity per DAS28 scores. The substantially shorter mean treatment duration of bDMARD therapy (4.7–34.5 weeks) in our study population versus that shown in other studies [23, 24] may be responsible for the suboptimal remission rates that we observed. The mean duration of treatment with etanercept, adalimumab, and infliximab in Italian patients with RA from the Italian Group for the Study of Early Arthritis registry was 3.1, 2.6, and 2.7 years, respectively [23]. Patients with RA from the Swiss Clinical Quality Management in Rheumatic Diseases registry received anti-TNF therapy for a median duration of 37 months [24]. The possible reason for the short treatment duration in the Chinese patients in the current study may be associated with poor socioeconomic condition, poor patient compliance, and the limitation of cross-sectional study. bDMARDs are relatively expensive and are not covered by the national health insurance reimbursement policies in China.

This study demonstrated that the proportion of remission + LDA was the highest in patients with ≥12 months of treatment, indicating that long-time treatment maximizes the benefits for patients. These results are in line with those of a previous 4-year follow-up study of infliximab therapy in refractory RA patients, which showed that longer duration of infliximab therapy resulted in lower DAS28 score [25]. Nam et al. found that maintenance of clinical responses was higher with bDMARD continuation [26]. Thus, our results suggest that, to achieve the best treatment outcome, patients with RA should continue bDMARD therapy for longer than 12 months. Furthermore, this study revealed that the remission rate in Chinese patients receiving bDMARDs was 12.6 % (DAS28), 5.4 % (CDAI), and 3.5 % (SDAI). In a multicenter cross-sectional study of RA in Chinese patients receiving csDMARDs, bDMARDs, and/or glucocorticoids, Wang et al. found the remission rate to be 8.6 % (DAS28), 8.2 % (CDAI), 8.4 % (SDAI), and 6.8 % (Boolean) [27]. In Qatari, the remission rate of patients with RA receiving bDMARDs and/or csDMARDs was 49 % by DAS28 score [28]. In the QUEST-RA study including patients with RA from 25 mostly European countries, remission rate was 13.8 % and 19.6 % by CDAI and DAS28, respectively [29]. These results indicate that treatment for Chinese patients with RA is insufficient. Further, the inhibitory costs of biologics ($15,000–$25,000 per patient per year) [30] present challenges in developing countries [31] and may result in early treatment discontinuation or dose reduction, resulting in flaring of the disease. While the guidelines do not recommend treatment discontinuation, “step-down” or tapering strategies by careful dose reduction or injection spacing can maintain a disease-free status in patients who have achieved remission or low disease activity [32]. Studies have shown that patients in remission after bDMARD mono or combination therapy continued to maintain their remission status after careful dose tapering or treatment discontinuation [33, 34]. Further, in order to maintain the alleviating effect, the dose of traditional DMARDS may be increased or treatment options such as double and triple combination treatment may be prescribed. Therapy can be reinitiated when necessary, making this approach desirable from a safety viewpoint and curbing unnecessary healthcare expenditure. Careful patient selection based on clinical and practical considerations may also improve outcomes with continuous bDMARD use in the developing world.

The current RA management regimen in China should be optimized to allow patients to benefit from more aggressive treatment. Given the comparable efficacy and cost-effectiveness of triple DMARD combinations in comparison with bDMARDs and methotrexate [35, 36], further studies are needed to assess the use of triple DMARD combination therapy in comparison with biologics in China.

Although the 2013 EULAR guidelines for RA management recommend bDMARDs to be used concomitantly with csDMARDs [10], the 2015 ACR guidelines take monotherapy into consideration [37] and bDMARDs monotherapy is also used in clinical practice as needed [12]. In some studies, bDMARD monotherapy has been shown to be as effective as the combination therapy of csDMARDs and bDMARDs. In terms of DAS28 score, ACR responses, and swollen and tender joint counts, tocilizumab and methotrexate combination therapy was not superior to tocilizumab monotherapy [13, 38]. Consistently, the current study also demonstrated that DAS28 score in patients receiving tocilizumab monotherapy was similar to that in patients receiving tocilizumab and csDMARD combination therapy. In the current study, although the average DAS28 score was significantly higher in patients receiving bDMARD monotherapy than in patients receiving combination therapy, the rate of reaching treatment target was similar in patients receiving monotherapy versus combination therapy. Interestingly, the current study found that patients with tocilizumab monotherapy showed significantly lower DAS28 score and higher proportion of reaching treatment target than patients receiving other types of bDMARD monotherapy. These findings suggest that tocilizumab monotherapy may be a promising option for patients with RA. Simplification and optimization of the treatment for RA are beneficial for both patients and physicians.

This cross-sectional study design presents a “snapshot” of bDMARD usage in Chinese patients with RA. It is difficult to infer causal relationships between bDMARD usage pattern and disease activity of RA, although longer duration of bDMARD therapy was associated with lower DAS28 score and higher rate of achievement of treatment goal through ad hoc analysis. Confounding factors that might affect the relationship between duration of bDMARD therapy and disease activity, such as the disease status prior to receiving bDMARD therapy and other concomitant therapies, were not considered in this study.

In conclusion, the usage of bDMARDs in Chinese patients with RA is in accordance with the global recommendations despite the short treatment duration. The bDMARDs are commonly used in combination with csDMARDs. Longer duration of bDMARD therapy appears to be associated with lower DAS28 scores and higher proportion of achievement of treatment target. These results should be kept in mind by the clinicians while treating patients with RA who do not achieve the treatment goal within 3 to 6 months of therapy. To achieve the best treatment outcome, patients with RA should continue bDMARD therapy for longer than 12 months. Our results provide a strong evidence base for the government to make decisions for the benefit of a greater number of patients with RA. Tocilizumab monotherapy may be a promising option for patients with RA. Further prospective studies are needed to assess the impact of bDMARD usage on RA remission among the Chinese population.
